# Monoallelic deletion of the microRNA biogenesis gene *Dgcr8 *produces deficits in the development of excitatory synaptic transmission in the prefrontal cortex

**DOI:** 10.1186/1749-8104-6-11

**Published:** 2011-04-05

**Authors:** Claude M Schofield, Ruby Hsu, Alison J Barker, Caitlyn C Gertz, Robert Blelloch, Erik M Ullian

**Affiliations:** 1 Department of Ophthalmology, University of California, San Francisco, San Francisco, CA 94143, USA; 2 Department of Physiology, University of California, San Francisco, San Francisco, CA 94143, USA; 3 Department of Urology, University of California, San Francisco, San Francisco, CA 94143, USA

## Abstract

**Background:**

Neuronal phenotypes associated with hemizygosity of individual genes within the 22q11.2 deletion syndrome locus hold potential towards understanding the pathogenesis of schizophrenia and autism. Included among these genes is *Dgcr8*, which encodes an RNA-binding protein required for microRNA biogenesis. Dgcr8 haploinsufficient mice (Dgcr8+/-) have reduced expression of microRNAs in brain and display cognitive deficits, but how microRNA deficiency affects the development and function of neurons in the cerebral cortex is not fully understood.

**Results:**

In this study, we show that Dgcr8+/- mice display reduced expression of a subset of microRNAs in the prefrontal cortex, a deficit that emerges over postnatal development. Layer V pyramidal neurons in the medial prefrontal cortex of Dgcr8+/- mice have altered electrical properties, decreased complexity of basal dendrites, and reduced excitatory synaptic transmission.

**Conclusions:**

These findings demonstrate that precise microRNA expression is critical for the postnatal development of prefrontal cortical circuitry. Similar defects in neuronal maturation resulting from microRNA deficiency could represent endophenotypes of certain neuropsychiatric diseases of developmental onset.

## Background

The cerebral cortex is the region in the mammalian brain associated with higher order cognitive and sensory processing. Integral to cortical function are interconnected networks of excitatory and inhibitory neurons, whose activity and connectivity emerge and strengthen through embryonic and postnatal development. Cortical neuron development requires the coordinated expression of specific genes that shape important physiological and structural properties, including dendritic arborization and the formation of GABAergic and glutamatergic synapses. Misregulation of these developmental processes has the potential to alter neuronal function and disrupt cortical circuitry, which may produce cognitive deficits that are a hallmark of certain mental disorders, including autism and schizophrenia. Accordingly, fully understanding the total complement of biological pathways that regulate the functional development of cortical neurons is of paramount importance.

microRNAs (miRNAs) are a recently described class of small (approximately 22-nucleotide) non-coding RNAs that function in a regulatory capacity. miRNAs can powerfully control gene expression by binding to complementary sequences within the 3' untranslated region of target messenger RNAs, where they lead to the suppression of translation or mRNA degradation [1,2]. miRNA biogenesis requires a series of sequential enzymatic processing reactions, and key among these is DGCR8 (DiGeorge syndrome critical region gene 8), an RNA-binding protein that partners with the RNase III enzyme Drosha to cleave initially long primary-miRNA transcripts into approximately 70-nucleotide stem-loop precursor-miRNAs [3]. These in turn are exported from the nucleus and further processed by Dicer into mature, functional miRNAs. The activity of DGCR8 can control cellular levels of miRNAs and it has been demonstrated that haploinsufficiency or ablation of DGCR8 protein can cause a 'bottleneck' in miRNA production, leading to significant increases in primary-microRNAs and concomitant decreases in functional, mature miRNAs [4,5]. Consequently, *in vivo *knockdown of DGCR8 can be used as a molecular tool to specifically inhibit miRNA synthesis and thus reveal miRNA-dependent physiological processes.

miRNAs are abundantly expressed in the mammalian brain and several reports have described regulatory roles for individual miRNAs in important functional processes in neurons [6-10]. However, the implications of targeted genetic deletion of specific miRNA biogenic proteins *in vivo *on the development and function of neurons in the cerebral cortex are minimally understood. Such studies would be important to test the intriguing hypothesis that miRNA dysregulation might perturb neural function and contribute to the pathogenesis of some neuropsychiatric diseases [11,12]. Human genetic studies of schizophrenia [13,14] and the 22q11.2 deletion syndrome (22q11DS), a chromosomal microdeletion that confers high susceptibility for schizophrenia and autism [15], suggest a possible association with miRNA misregulation. These findings would postulate that animal models of miRNA dysfunction, especially defects in biogenesis, could potentially display cellular phenotypes relevant to mental illness. To probe this link, we used a multidisciplinary approach to investigate the function and structure of pyramidal neurons in the prefrontal cortex of *Dgcr8 *heterozygous mice (Dgcr8+/-). We find that Dgcr8+/- mice display reduced expression of a subset of miRNAs in the prefrontal cortex, a deficiency that emerges over postnatal development. Layer V (L5) pyramidal neurons of Dgcr8+/- mice show changes in their intrinsic electrical properties, deficits in the complexity of basal dendrites and impaired development of excitatory synaptic transmission.

## Results

**Figure 1:**
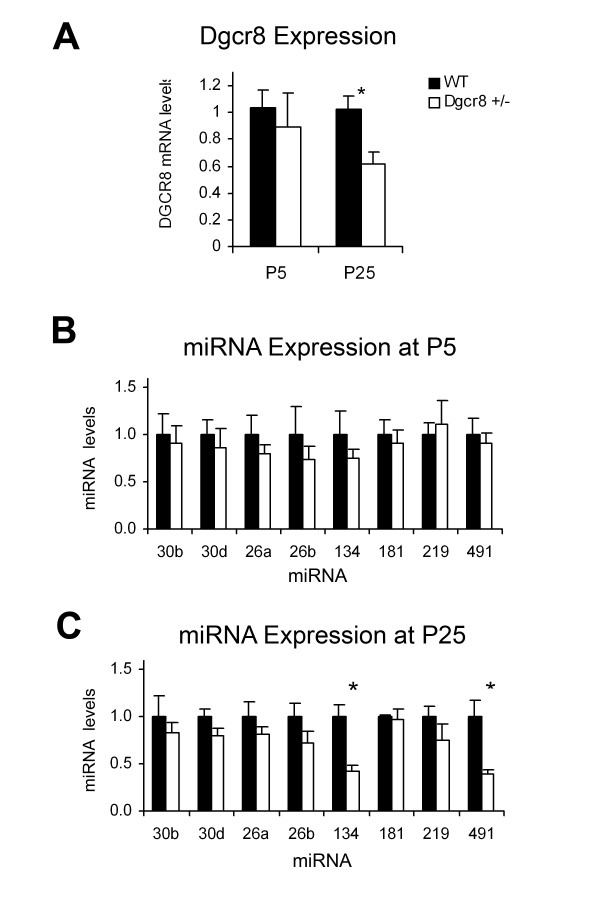
**Reduced miRNA expression in Dgcr8+/- mice**. *Dgcr8 *mRNA and miRNA expression in the prefrontal cortex of WT (*n *= 5) and Dgcr8+/- (*n *= 5) animals at postnatal day (P)5 and P25. **(A) **Quantitative PCR showing a significant reduction in *Dgcr8 *mRNA levels in the heterozygous animal at P25 but not at P5. **(B,C) **Quantitative PCR of a selected panel of miRNAs showing significant reductions of specific miRNAs at P25 but not at P5. Values are shown as relative expression compared to WT. Bars represent mean ± standard error; *P < 0.01.

Dgcr8+/- mice are viable, represented at normal birth frequencies in litters, and display gross brain morphologies that are indistinguishable from wild type (WT). We initially sought to confirm that *Dgcr8 *heterozygosity leads to reduced expression of miRNAs in the cortex, as previously reported in a uniquely generated Dgcr8+/- mouse line [5]. To assess this, we examined mRNA and miRNA levels in frontal cortex brain lysates from control WT and Dgcr8+/- mice during postnatal development (Figure 1A). Surprisingly, at postnatal day (P)5 Dgcr8+/- frontal cortices showed no significant changes in *Dgcr8 *mRNA levels assessed with quantitative PCR (qPCR). qPCR was also used to examine the expression of a panel of select brain-enriched miRNAs and these were similarly unaffected (Figure 1B). In contrast, by P25, *Dgcr8 *mRNA was significantly downregulated by 40 ± 9% in Dgcr8+/- cortex (*P *= 0.01; Figure 1A). qPCR established that reduced expression of *Dgcr8 *mRNA in Dgcr8+/-mice at P25 resulted in the reduced expression of a subset of miRNAs (miR-134, 57 ± 6%, *P *= 0.001; miR-491, 61 ± 6%, *P *= 0.004; Figure 1C). These data demonstrate that *Dgcr8 *heterozygosity leads to reduced biogenesis of miRNAs in cortex; however, this deficiency is not displayed in neonatal mice but rather emerges over development.

**Figure 2:**
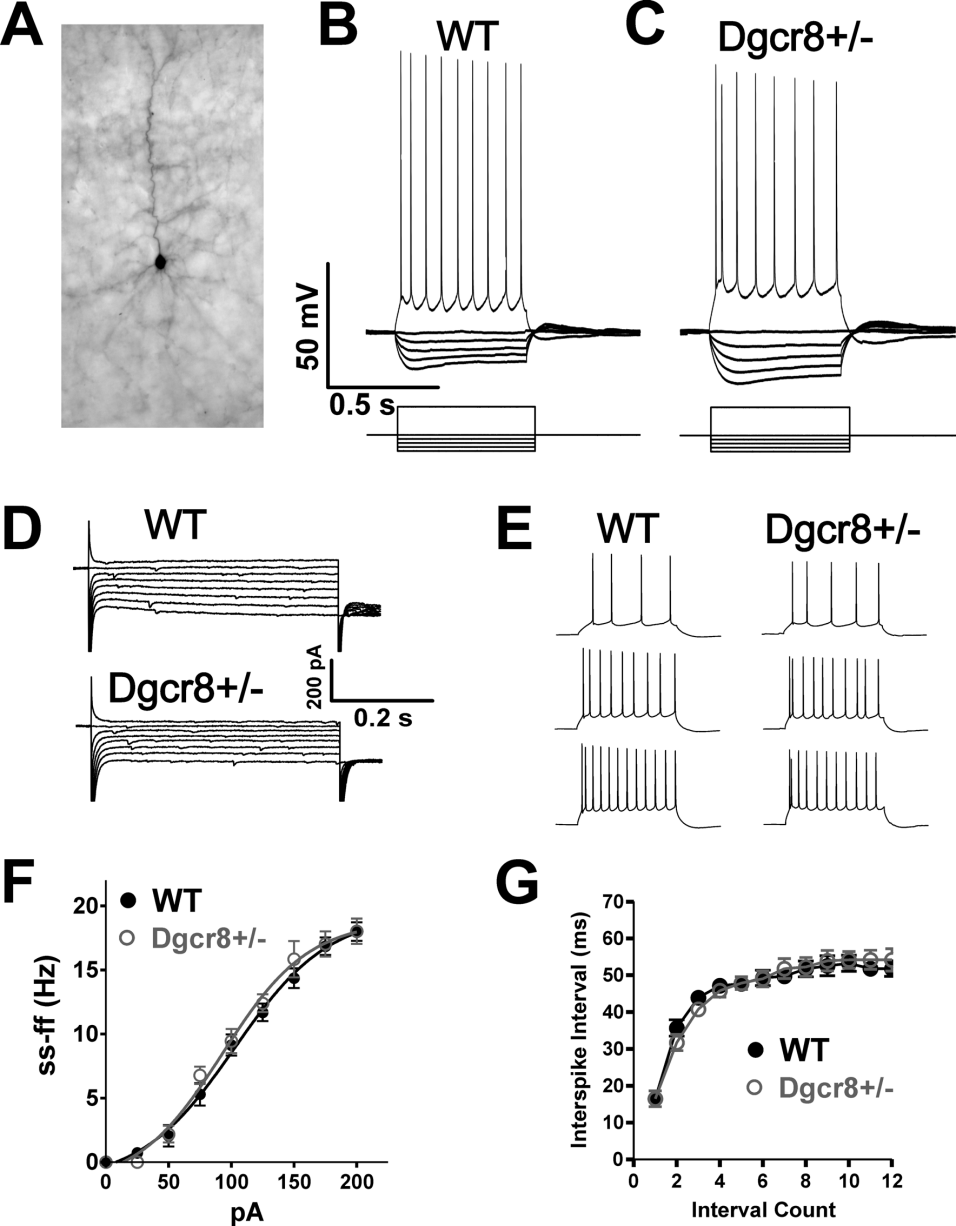
**Altered electrical properties, but normal spike firing capabilities of pyramidal neurons in Dgcr8+/- mice**. **(A) **Magnified image (20×) of a neurobiotin-labeled L5 mPFC pyramidal neuron filled during whole-cell recording. **(B,C) **Representative current-clamp recordings from mPFC L5 pyramidal neurons from (B) WT and (C) Dgcr8+/- mice show stereotyped pyramidal neuron firing patterns in both genotypes in response to a series of hyperpolarizing (-200 to -25 pA) and depolarizing (+100 pA) current injections. **(D) **I-V relationships were assessed in voltage-clamp mode through a series of 5-mV steps and demonstrate increased input resistance (*R*_in_) in Dgcr8+/- neurons. **(E) **Action potential firing responses in WT and Dgcr8+/- neurons evoked by +75, +125 and +175 pA current injections. **(F) **The plot of the steady-state firing frequency (ss-ff) as a function of current intensity shows no changes in the input-output response between genotypes (WT *n *= 17; Dgcr8+/- *n *= 11). **(G) **Plot of the interspike interval between spikes of a train of action potentials evoked by +200 pA current injection demonstrates no changes in firing capabilities (WT *n *= 17; Dgcr8+/- *n *= 11).

In order to investigate the functional consequences of miRNA deficiency in the brain, we examined the electrophysiological properties of cortical neurons in Dgcr8+/- mice by performing voltage and current clamp recordings on L5 pyramidal neurons in the medial prefrontal cortex (mPFC). These neurons are identifiable by their large soma, prominent apical dendrite (Figure 2A) and stereotyped electrophysiological properties, including regular spiking activity with minimal accommodation (Figure 2B). We initially characterized the passive membrane properties and action potential firing capabilities of L5 pyramidal neurons. Input resistance (*R*_in_) was measured through the I-V plot of whole-cell current responses to a series of 5 mV voltage steps (Figure 2D) and this parameter was significantly increased by approximately 30% in Dgcr8+/- neurons compared to WT (WT *R*_in _= 151 ± 7 MΩ, n = 22 cells; Dgcr8+/- *R*_in _= 195 ± 10 MΩ, *n *= 20 cells; *P *= 0.002). Conversely, measurement of the whole-cell capacitance (C_c_) showed that this value was significantly decreased in Dgcr8+/- L5 pyramidal neurons (WT C_c _= 111 ± 4 pF, *n *= 24 cells; Dgcr8+/- C_c _= 93 ± 4 pF, *n *= 22 cells; *P *= 0.004). These changes in passive electrical properties might be attributable to changes in specific membrane conductance or leak currents, so we examined the membrane time constants (τ_m_) and resting membrane potential of L5 pyramidal neurons. τ_m _values were determined by the single exponential fit of the time course of the membrane voltage response to a -25 pA current step, and these values were similar between genotypes (WT τ_m _= 34 ± 2 ms, *n *= 22 cells; Dgcr8+/- τ_m _= 36 ± 1 ms, *n *= 27 cells; *P *= 0.31). The resting membrane potential was also unchanged (WT V_m _= -62 ± 2 mV, *n *= 18 cells; Dgcr8+/- V_m _= -61 ± 1 mV, *n *= 22 cells; *P *= 0.85). Together, these data show that Dgcr8+/- neurons display altered whole-cell electrical properties, without observable changes to specific membrane properties or leak conductances.

Changes in passive electrical properties might alter neuronal excitability, so we next examined the spike firing capabilities of L5 pyramidal neurons in WT and Dgcr8+/- mice using current-clamp. We first probed neuronal excitability by measuring the minimal current required to elicit an action potential (rheobase current) and this value was not significantly changed (WT = 67 ± 3 pA, *n *= 22 cells; Dgcr8+/- = 60 ± 3 ms, *n *= 19 cells; *P *= 0.08). The threshold for action potential was also not altered (WT V_Thr _= -39 ± 6 mV, *n *= 23 cells; Dgcr8+/- V_Thr _= -40 ± 1 mV, *n *= 20 cells). Next we examined action potential firing rates of WT and Dgcr8+/- neurons (Figure 2E). The input-output relationship was assessed by measuring the neuron's steady-state firing frequency rate as a function of amplitude of injected current (Figure 2F) and this plot was found to be similar between genotypes. Likewise, examination of the interspike interval of a train of spikes elicited by +200 pA demonstrated indistinguishable firing patterns (Figure 2G). Together, these data show that despite alterations in passive electrical properties, the excitability and spike firing capabilities of Dgcr8+/- pyramidal neurons is unaffected by reduced miRNA expression.

**Figure 3:**
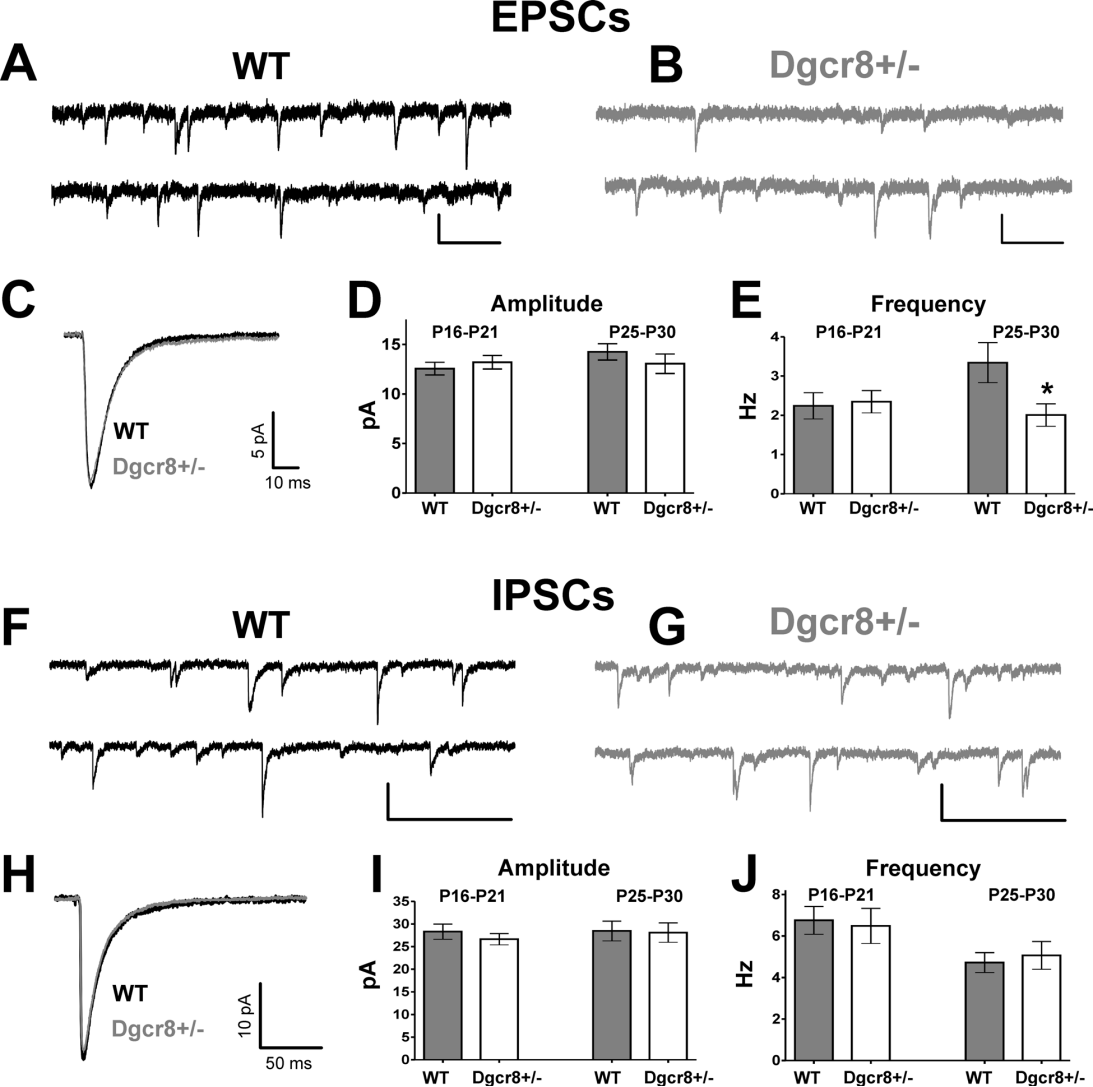
**Maturation-dependent reduction in excitatory postsynaptic current frequency in Dgcr8+/- mPFC**. **(A,B) **Representative excitatory postsynaptic current (EPSC) recordings from L5 pyramidal neurons from (A) WT and (B) Dgcr8+/- mice. Scale bar = 20 pA, 200 ms. **(C) **Mean EPSC responses (average of >50 isolated events) from individual WT (black) and Dgcr8+/- (grey) neurons superimposed on the same scale illustrate no changes in EPSC amplitude or kinetics. **(D,E) **Summary of EPSC parameters from WT (*n *= 15) and Dgcr8+/- (*n *= 18) neurons reveals a maturation-dependent reduction in EPSC frequency from between P16 and P21 to between P25 and P30. **(F,G) **Inhibitory postsynaptic current (IPSC) recordings from (F) WT and (G) Dgcr8+/- pyramidal neurons. Scale bar = 50 pA by 500 ms. **(H) **Mean IPSC responses (average of >50 isolated events) from individual WT (black) and Dgcr8+/- (grey) neurons; **(I,J) **summary graphs of amplitudes and frequencies demonstrate no changes in inhibitory synaptic transmission at these periods. Bars represent mean ± standard error; *P < 0.01.

Because miRNAs can exert powerful regulatory control over translation, we hypothesized that neuronal miRNA deficiency could consequently alter translation-dependent processes that occur during cortical development, including synapse formation and function. To assess this, we used whole-cell patch-clamp electrophysiology to examine synaptic currents in mPFC L5 pyramidal neurons in WT and Dgcr8+/- mice. We analyzed spontaneous excitatory postsynaptic currents (EPSCs; Figure 3A, B) and spontaneous inhibitory postsynaptic currents (IPSCs; Figure 3F, G) at two time periods during postnatal development. In recordings from P16 to P21 mice (WT *n *= 15 cells; Dgcr8+/- *n *= 14 cells) we found no significant changes in EPSC event parameters, including amplitude (WT = 13 ± 1 pA; Dgcr8+/- = 13 ± 1 pA; Figure 3D) or frequency (WT = 2.2 ± 0.3 Hz; Dgcr8+/- = 2.3 ± 0.3 Hz; Figure 3E). Similarly, IPSC event amplitude (WT = 30 ± 1 pA; Dgcr8+/- = 27 ± 1 pA; Figure 3I) and frequency (WT = 6.8 ± 0.7 Hz; Dgcr8+/- = 6.5 ± 0.8 Hz; Figure 3J) were unaffected during the P16 to P21 period. However, when we examined synaptic currents in older P25 to P30 mice (WT *n *= 15 cells; Dgcr8+/- *n *= 18 cells) we found a significant decrease in the frequency of EPSCs (WT = 3.3 ± 0.5 Hz; Dgcr8+/- = 2.0 ± 0.3 Hz; *P *= 0.02; Figure 3E) without changes in EPSC amplitude (WT = 14.2 ± 0.8 pA; Dgcr8+/- = 13.6 ± 0.8 pA; Figure 3D), EPSC kinetics (Figure 3C) or changes in IPSC amplitude (WT = 28 ± 2 pA; Dgcr8+/- = 28 ± 2 pA; Figure 3I), IPSC frequency (WT = 4.7 ± 0.5 Hz; Dgcr8+/- = 5.1 ± 0.7 Hz; Figure 3J) or IPSC kinetics (Figure 3H). To further probe this result, we also examined the 'miniature' EPSC (mEPSC) event population in P25 to P30 mice. Similar to the spontaneous event data, mEPSC frequency was reduced in Dgcr8+/- neurons (WT = 1.9 ± 0.4 Hz; Dgcr8+/- = 0.9 ± 0.1 Hz; *P *= 0.02; Additional file 1) without changes to mEPSC amplitude (WT = 11 ± 1 pA; Dgcr8+/- = 10 ± 1 pA). Lastly, we performed some additional recordings at 33 to 34°C to determine if these deficits persist near physiological temperatures. Elevation of temperatures increased the frequency and amplitude of mEPSC events in both genotypes, and consistent with our previous findings, the deficit in EPSC frequency persisted (WT = 3.8 ± 1.0 Hz; Dgcr8+/- = 2.9 ± 0.9 Hz). Summarily, in WT mPFC we observed endogenous changes in synaptic transmission during maturation, with increased EPSC frequency and decreased IPSC frequency occurring between 3 and 4 postnatal weeks. miRNA deficiency abolished this normal developmental increase in EPSC frequency while not effecting IPSCs, leading to a shift in the balance of excitation/inhibition at P25 to P30. This observed reduction in EPSC frequency in the absence of changes in IPSCs indicates that cortical miRNA deficiency alters the balance of spontaneous synaptic transmission.

**Figure 4:**
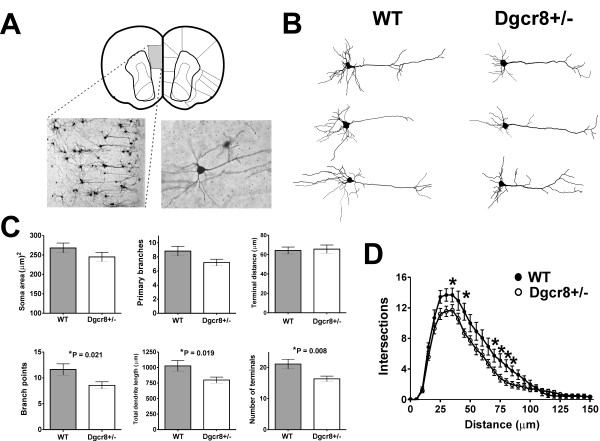
**Reduced basal dendritic complexity of L5 pyramidal neurons in Dgcr8+/- mice**. Golgi-cox staining of mPFC from WT and Dgcr8+/- mice at P25. **(A) **Diagram of coronal section of mouse mPFC delineating the area of study and representative 10× and 40× magnification images of L5 pyramidal neurons. **(B) **Traces from Neurolucida reconstructions are shown for three representative WT and Dgcr8+/- neurons. **(C) **Summaries of morphometric data from WT (*n *= 16 neurons) and Dgcr8+/- mice (*n *= 20 neurons). Cell body area, number of primary basal dendrites, and the average basal terminal distance from soma were not different between genotypes. Statistically significant decreases were observed in the number of basal dendrite branch points, total dendritic length, and number of terminals. Bars represent mean ± standard error; *P < 0.05. **(D) **Scholl analysis of basal dendrites shows reduced complexity in Dgcr8+/- neurons; *P < 0.05.

One possible explanation for the neurophysiological changes observed in Dgcr8+/- mice is altered neuron morphology. Input resistance and whole-cell capacitance are functions of membrane area and the primary sites of excitatory synapses onto pyramidal neurons are the dendrites. Accordingly, a decrease in the number of basal dendrites in Dgcr8+/- pyramidal neurons would reduce both membrane area and the number of postsynaptic sites and potentially account for the observed phenotypes. To assess this, we performed Golgi staining (Figure 4A) and complete three-dimensional reconstructions of L5 pyramidal neurons (Figure 4B) from WT (*n *= 16 cells from 5 animals) and Dgcr8+/- mice (*n *= 20 cells from 5 animals). Morphometric analysis (Figure 4C) of the soma revealed no changes to shape or soma area (WT = 268 ± 13 μm^2^; Dgcr8+/- = 245 ± 12 μm^2^). Likewise, analysis of the apical dendritic branch showed that these structures remained unaffected in Dgcr8+/- neurons as we found that the number of apical dendrite branch points (WT = 6 ± 1; Dgcr8+/- = 6 ± 1) and the apical dendrite terminal distance from soma (WT = 297 ± 21 μm; Dgcr8+/- = 319 ± 15 μm) were unaltered. However, we found multiple changes to the structure of basal dendrites in Dgcr8+/- mice. Scholl analysis (Figure 4D) revealed decreased branch complexity in Dgcr8+/- and this was attributable to a decreased number of dendritic branch points (WT = 12 ± 1; Dgcr8+/- = 9 ± 1; *P *= 0.02) resulting in a decreased total dendritic length (WT = 1,026 ± 86 μm; Dgcr8+/- = 798 ± 49 μm; *P *= 0.02). Lastly, we surveyed dendritic spines and found no differences in spine morphology between genotypes (Additional file 2) and the spine density on second order branches of the basal dendrites was unchanged (WT = 2.7 ± 0.3 spines/10 μm; Dgcr8+/- = 3.1 ± 0.3 spines/10 μm). Summarily, these data describe a specific morphological deficit in the branching and complexity of basal dendrites of L5 mPFC pyramidal neurons in Dgcr8+/- mice. During postnatal maturation, mPFC basal dendrites undergo elaboration and outgrowth that coincides with the development of the intrinsic electrical properties of pyramidal neurons [16]. The deficit in basal dendritic branching in Dgcr8+/- mice is consistent with a perturbation to this developmental process and can provide a potential mechanistic explanation for the neurophysiological phenotypes that we describe.

## Discussion

Mouse models of 22q11DS display behavioral deficits [17] and cortical abnormalities [18]. The finding that Dgcr8+/- mice display key behavioral deficits associated with 22q11DS [5] would indicate that Dgcr8 heterozygosity is sufficient to produce some of the neural deficits that underlie the 22q11DS. The results of the present study can potentially elucidate the neuronal basis of deficits of miRNA-dependent origin in the mPFC. We find that mice heterozygous for Dgcr8 display reduced expression of a subset of miRNAs in the forebrain. These effects are not observed in neonatal mice but rather emerge over postnatal development during the time period of pyramidal neuron maturation [16]. Reduced miRNA biogenesis in Dgcr8+/- mice coincides with specific neurophysiological deficits, including changes in the passive electrical properties and reduced frequency of EPSC events later during postnatal development. Morphometric analysis of pyramidal neurons revealed reduced complexity, length and branching of the basal dendrites, a finding consistent with the observed electrophysiological changes. These results demonstrate an essential role for Dgcr8-dependent miRNA synthesis in the maturation of pyramidal neurons during postnatal development and may provide a mechanistic explanation for the late developmental onset of deficits in 22q11DS.

How can these described cellular changes in Dgcr8+/- mice lead to deficits in behavior? Pyramidal neurons in the mPFC form recurrent excitatory synapses within L5 and the unique connectivity and strength of these synapses sustains intrinsic L5 excitability and persistent activity displayed during working memory [19]. The primary site of pyramidal neuron recurrent synapses is on the basal dendrites and, consequently, the reduced dendritic branching described in Dgcr8+/- mice could specifically impair these recurrent connections, leading to altered mPFC network activity. Consistent with the notion of altered connectivity are *in vivo *data from 22q11DS mice that demonstrated impaired synchronous electrical activity between the hippocampus and mPFC [20]. Interestingly, this same study also reported no differences in the firing rate of prefrontal cortex neurons, which is consistent with the current-clamp data we report here, as well as an additional study that found no differences in the firing rates of CA1 pyramidal neurons in 22q11DS mice [21]. Together, these indicate that the cellular deficits underlying 22q11DS are likely of synaptic origin rather than changes to intrinsic spike firing capabilities.

In probing haploinsufficiency we have uncovered a clear developmental progression that shows normal Dgcr8 and miRNA expression in P5 Dgcr8+/- cortex but reduced expression by P25. These data would suggest that during the crucial period for neurogenesis and differentiation, monoallelic loss of Dgcr8 is compensated, resulting in normal miRNA biogenesis in brain. This would explain the absence of a severe phenotype in Dgcr8+/- cortex, in contrast to the defects in cortical lamination, disrupted morphogenesis and widespread neuronal apoptosis observed as a consequence of conditional embryonic forebrain deletion of Dicer [22,23]. Dosage compensation has also been reported for the 22q11DS gene *Ufd1l*, although this occurs through translational, rather than transcriptional, regulation [24]. Conversely, in our case, during postnatal maturation Dgcr8 expression levels may enter a dynamic range to where monoallelic loss can produce haploinsufficiency and decreased miRNA expression, which in turn may dysregulate translational-dependent processes that occur during this crucial period, including dendritogenesis and synaptogenesis. Although these data provide a compelling correlation between brain miRNA expression and circuit development during the postnatal period, we cannot exclude that miRNAs are reduced in brain during the embryonic period in Dgcr8+/- mice and that such embryonic deficiencies are ultimately causal of the electrophysiological and synaptic deficits at P25. We also cannot dismiss the possibility that miRNA deficiency in non-brain tissues could produce systemic physiological changes that subsequently cause neuronal deficits. Still, in light of our findings, Dgcr8+/- mice may be useful to probe whether loss of compensatory mechanisms in regulating neuronal miRNA expression during development may contribute to any of the emergent phenotypes of schizophrenia and autism.

To our knowledge, this is the first study to extensively characterize the electrophysiological properties of miRNA-deficient neurons in the acute brain slice. Accordingly, these data have implications for understanding miRNA-dependent functional processes in neurons. One remaining question is the identity of the miRNA(s) whose haploinsufficiency can account for the cellular phenotypes characterized in this report. *In vitro *targeting studies in neuronal culture systems have implicated miR-132 in neurite sprouting [25] and the miR-379-410 cluster (which includes miR-134) in activity-dependent dendritic outgrowth [26]. However, as Dgcr8 is required for all *de novo *miRNA synthesis and the total number of brain-enriched miRNAs is likely >300 [27], a direct link between a single miRNA species and genuine *in vivo *mRNA target leading to these observed deficits is dubious. A more likely scenario is that reduced expression of a subset of miRNAs in Dgcr8+/- cortex leads to impairments in the capacity of neurons to 'fine-tune' expression of several target mRNAs, leading to increased expression of multiple proteins that produce the deficits in pyramidal neurons described here. To ascertain this, future development of methodology that can deliver miRNAs into brain and rescue precise levels of miRNA expression in pyramidal neurons and reverse the phenotypes in Dgcr8+/- mice will be necessary. Such miRNA delivery techniques could also hold promise as the basis of novel therapeutics that restore brain miRNA levels in individuals with neuropsychiatric diseases attributable to miRNA deficiency.

## Materials and methods

### Animals

Dgcr8+/- mice were generated as described [4] and bred and maintained against a C57BL/6J background backcrossed for at least four generations. Mice were genotyped by PCR analysis of tail biopsies, and all experiments were approved by the UCSF Institutional Animal Care and Use Committee.

### Quantitative real-time PCR

The frontal cortex containing the prefrontal (medial and orbital regions) and motor cortex areas were microdissected out of P5 or P25 mice brains. Total RNA was isolated from these using Trizol extraction methodology. To evaluate gene expression, 100 ng of total RNA was used to generate cDNA using the Taqman Reverse Transcription Kit (Applied Biosystems, Carlsbad, CA, USA). qPCR was performed using SYBR GreenER qPCR SuperMix (Invitrogen, Carlsbad, CA, USA) on a CFX96 Real-Time System and a C1000 Thermal Cycler (Bio-Rad, Hercules, CA, USA). To evaluate miRNA expression, 50 ng of total RNA was reverse transcribed using the Taqman MicroRNA Reverse Transcription Kit (Applied Biosystems). miRNA qPCR was performed using the Taqman Universal PCR Master Mix (Applied Biosystems) and custom-designed Taqman probes (IDT DNA Technologies, Coralville, IA, USA) using techniques previously described [28]. The U6 snRNA was used as an internal control. All qPCR reactions were performed in triplicate and relative quantifications were calculated using the Pfaffl method [29].

### Slice preparation

Prefrontal cortical slices were prepared from WT and Dgcr8+/- littermate control mice of either sex, from two age-matched postnatal groups (P16 to P21 and P25 to P30). Animals were anesthetized with isoflurane and decapitated, and the whole brain was removed and transferred into ice-cold cutting solution containing (in mM): 75 sucrose, 87 NaCl, 25 glucose, 25 NaHCO_3_, 2.5 KCl, 1.25 NaH_2_PO_4_, 10 MgSO_4_, and 0.5 CaCl_2_, equilibrated with 95% O_2_/5% CO_2_. Coronal sections 250 to 350 μm thick were cut on a vibratome and then transferred into a 33°C incubation chamber with artificial cerebral spinal fluid (ACSF) containing (in mM): 125 NaCl, 25 NaHCO_3_, 2.5 KCl, 1.25 NaH_2_PO_4_, 2 MgCl_2_, 1 CaCl_2_, and 10 glucose, equilibrated with 95% O_2_/5% CO_2 _prior to recording.

### Electrophysiology

Whole-cell patch-clamp recordings were performed using a MultiClamp 700A amplifier (Molecular Devices, Sunnyvale, CA, USA) in a submerged bath recording chamber superfused with ACSF at a rate of 2 ml/minute. Recording electrodes were made of borosilicate glass and had a resistance of 2.5 to 4 MΩ when filled with intracellular solution, which contained (in mM): 110 K-gluconate, 20 KCl, 10 HEPES, 0.4 EGTA, 2 MgCl_2_; pH was 7.25 and osmolarity was adjusted to 280 to 290 mOsm with sucrose. During voltage clamp recordings, cells were clamped at -70 mV and spontaneous EPSCs were pharmacologically isolated by bath application of the GABA-A receptor antagonist bicuculline methiodide (10 μM, Tocris). For mEPSC recordings 1 μM tetrodotoxin (TTX, Ascent Scientific, Princeton, NJ, USA) was included in the ACSF. For recording spontaneous IPSCs the internal contained (in mM): 135 CsCl, 10 HEPES, 10 EGTA, 5 QX-314 and 2 MgCl_2 _and the external ACSF contained 20 μM DNQX (6,7-dinitroquinoxaline-2,3-dione) and 50 μM AP-5. L5 pyramidal neurons were visually identified based on morphology and position using a fixed-stage upright microscope (Nikon FN1) under 40× magnification and IR-DIC optics. Access resistance was monitored and cells were included for analysis only if the series resistance was <20 MΩ and the change of resistance was <25% over the course of the experiment. Data were acquired at 10 kHz using pClamp 10.2 (Molecular Devices) and filtered at 2 kHz. The liquid junction potential was 12.3 mV and atomically corrected for in pClamp. In some recordings, 0.2% Neurobiotin (Vector Labs, Burlingame, CA, USA) was included in the intracellular solution in the patch pipette. Afterwards, slices with labeled neurons were fixed in 4% paraformaldehyde in 0.1 M phosphate buffer overnight, then processed and stained according to protocol.

### Golgi staining and neuron reconstruction

Whole brains from Dgcr8+/- and WT littermate controls were removed at P25 and Golgi-cox impregnation and staining were performed according to protocol (FD Rapid Golgi Staining, FD Neurotechnologies, Ellicott City, MD, USA) Coronal sections of 120 μm were cut on a cryostat, mounted onto gelatin slides, cleared with ethanol and xylene and coverslipped. Three-dimensional reconstructions of neurons were performed blind to genotype at 40× brightfield magnifications using an Olympus BX-51 microscope equipped with a computer-controlled motorized stage and Neurolucida software (MBF Biosciences, Williston, VT, USA). For analysis, we only selected pyramidal cells positioned in L5 of the prelimbic and infralimbic regions of the medial prefrontal cortex from brain sections that were from similar coronal planes, as determined by size and position of the forceps minor corpus callosum. We excluded superficially positioned neurons to ensure that complete dendritic trees were intact.

## Abbreviations

22q11DS: 22q11.2 deletion syndrome; ACSF: artificial cerebral spinal fluid; EPSC: excitatory postsynaptic current; IPSC: inhibitory postsynaptic current; L5: layer V; mEPSC: miniature excitatory postsynaptic current; miRNA: microRNA; mPFC: medial prefrontal cortex; P: postnatal day; qPCR: quantitative PCR; WT: wild type.

## Competing interests

The authors declare that they have no competing interests.

## Authors' contributions

CMS designed and conducted the electrophysiology and Golgi experiments and wrote the paper. RH performed the qPCR and image analysis. AJB contributed to experiments and analysis. CCG contributed to experiments and analysis. RB generated the Dgcr8+/- mice. EMU supervised the study, participated in its design and coordination and helped edit the manuscript.

## Supplementary Material

Additional file 1 **mEPSC frequency is reduced in Dgcr8+/ neurons**. **(A,B) **mEPSC recordings from L5 pyramidal neurons from (A) WT and (B) Dgcr8+/- mice, ages P25 to P30. **(C) **Summary of mEPSC parameters averaged from >50 isolated events per cell, WT (*n *= 5) and Dgcr8+/- (*n *= 5), demonstrates reduced mEPSC frequency (WT = 1.9 ± 0.4 Hz; Dgcr8+/- = 0.9 ± 0.1 Hz; *P *= 0.02) and no changes to mEPSC amplitude (WT = 11 ± 1 pA; Dgcr8+/- = 10 ± 1 pA). Click here for file

Additional file 2 **Unaltered spines on Dgcr8+/-pyramidal neurons**. **(A,B)** 100× magnification images of spines on second order branches of basal dendrites from Golgi stained L5 pyramidal neurons from WT and Dgcr8+/- mPFC. **(C,D) **Summary graphs demonstrate no changes in spine length or spine width between genotypes (WT = 154 spines, Dgcr8+/- = 123 spines). **(E) **Summary graph shows no changes in spine density between WT and Dgcr8+/- (*n *= 25 dendritic branches from 5 animals per genotype). Click here for file
