# Supplementary figures and images for: Monoallelic deletion of the microRNA biogenesis gene Dgcr8 produces deficits in the development of excitatory synaptic transmission in the prefrontal cortex
Source: Neural Dev. 2011 Apr 5;6:11. doi: 10.1186/1749-8104-6-11 (PMC3082233; doi:10.1186/1749-8104-6-11)

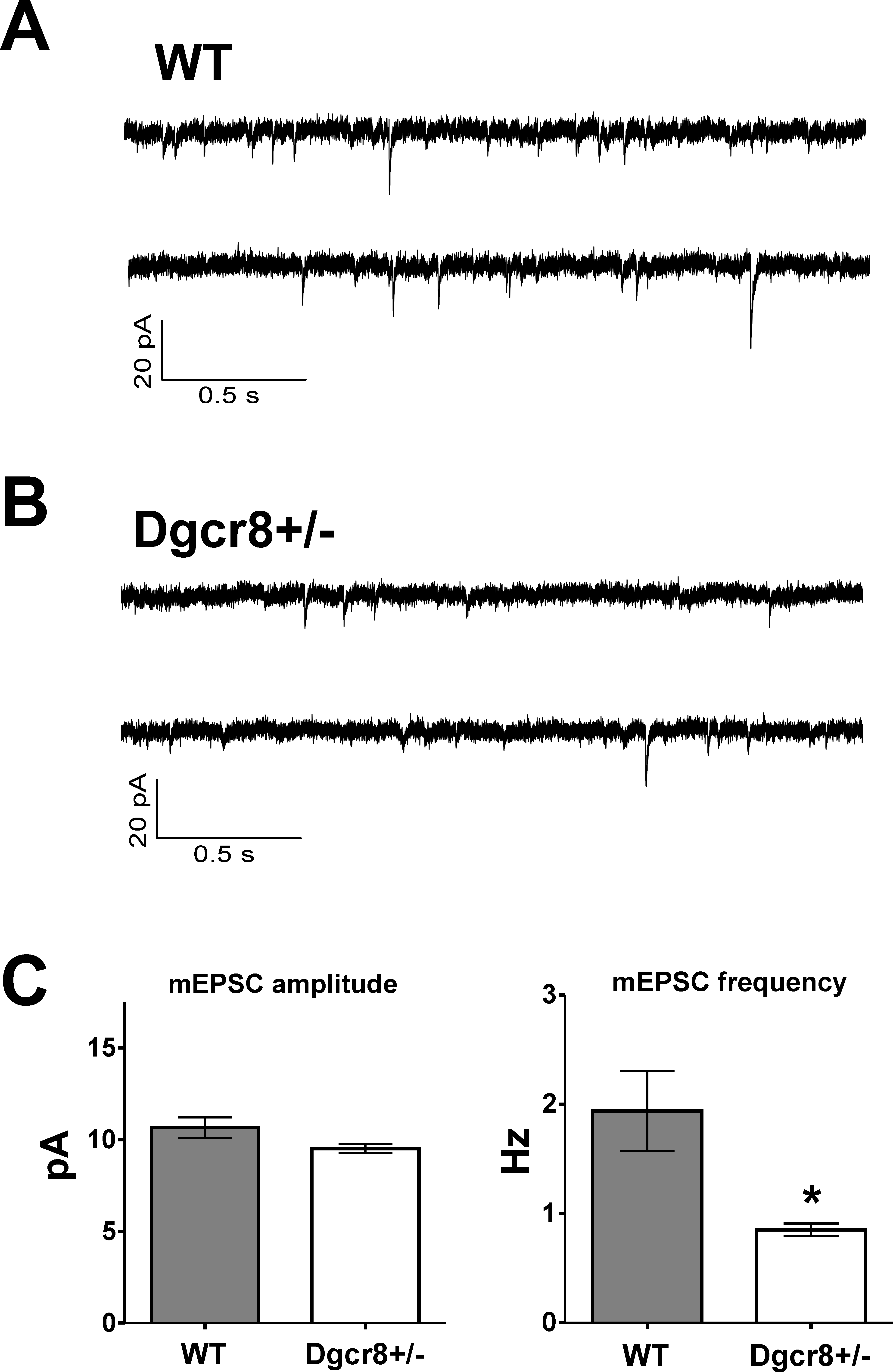

Supplement: Additional file 1 — mEPSC frequency is reduced in Dgcr8+/ neurons. (A,B) mEPSC recordings from L5 pyramidal neurons from (A) WT and (B) Dgcr8+/- mice, ages P25 to P30. (C) Summary of mEPSC parameters averaged from >50 isolated events per cell, WT (n = 5) and Dgcr8+/- (n = 5), demonstrates reduced mEPSC frequency (WT = 1.9 ± 0.4 Hz; Dgcr8+/- = 0.9 ± 0.1 Hz; P = 0.02) and no changes to mEPSC amplitude (WT = 11 ± 1 pA; Dgcr8+/- = 10 ± 1 pA). [file 1749-8104-6-11-S1.TIFF]

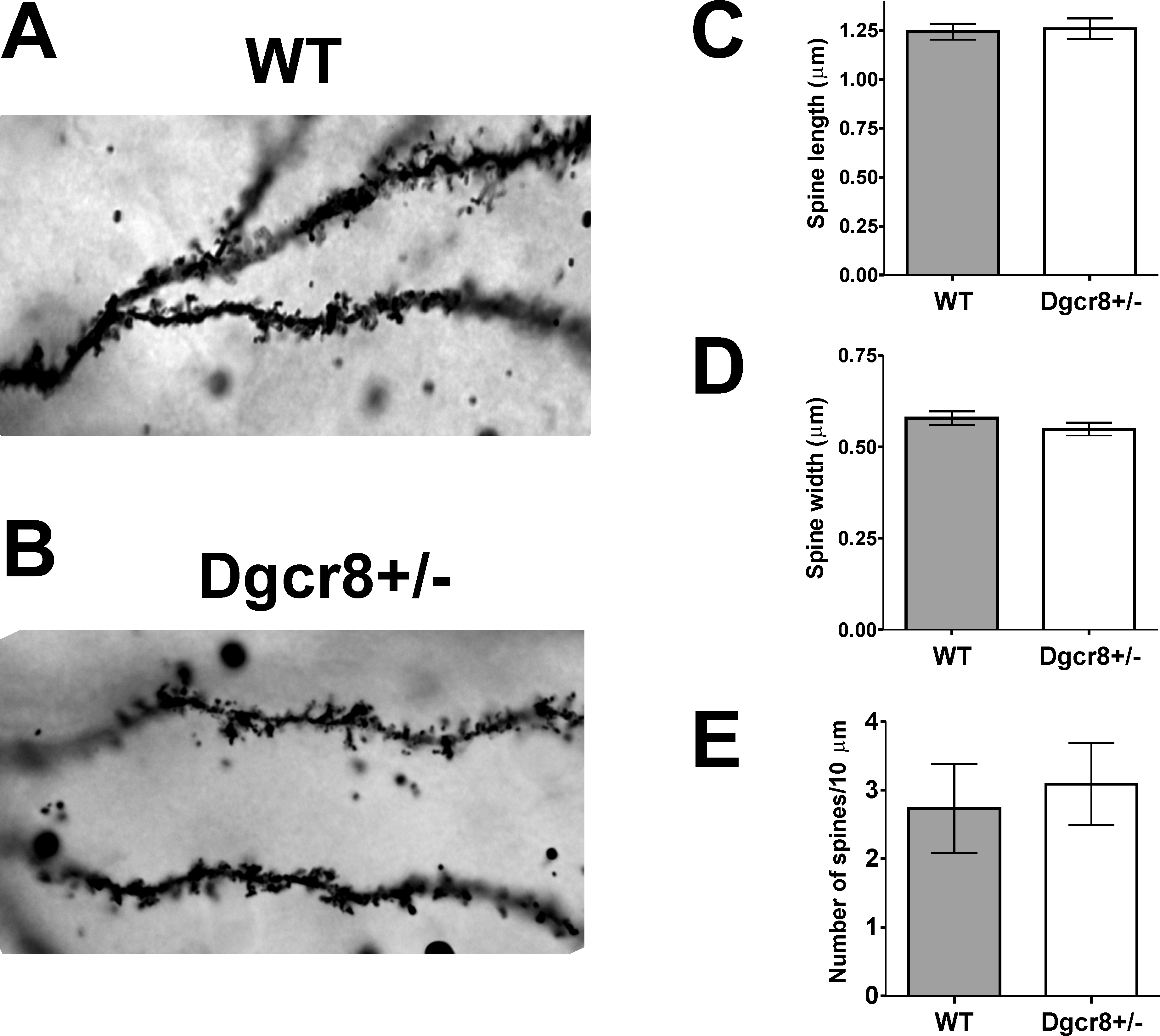

Supplement: Additional file 2 — Unaltered spines on Dgcr8+/-pyramidal neurons. (A,B) 100× magnification images of spines on second order branches of basal dendrites from Golgi stained L5 pyramidal neurons from WT and Dgcr8+/- mPFC. (C,D) Summary graphs demonstrate no changes in spine length or spine width between genotypes (WT = 154 spines, Dgcr8+/- = 123 spines). (E) Summary graph shows no changes in spine density between WT and Dgcr8+/- (n = 25 dendritic branches from 5 animals per genotype). [file 1749-8104-6-11-S2.TIFF]
